# The Influence of Sexual Identity on Sexual Harassment During Residency Training: A Survey Study

**DOI:** 10.5435/JAAOSGlobal-D-26-00084

**Published:** 2026-10-14

**Authors:** Nicole Infantino, Ashley Knebel, Mitchell J. Christiansen, Mary K. Mulcahey

**Affiliations:** From the College of Osteopathic Medicine, Touro University Nevada, Henderson, NV (Ms. Infantino); the Warren Alpert Medical School, Brown University, Providence, RI (Ms. Knebel); and the Department of Orthopedic Surgery and Rehabilitation, Loyola University Medical Center, Maywood, IL (Mr. Christiansen and Dr. Mulcahey).

## Abstract

**Introduction:**

Representation of diverse populations within orthopaedic surgery lags behind other specialties. Underrepresented minority orthopaedic surgeons report workplace discrimination based on race and sexual orientation; however, the influence of intersecting underrepresented minority identities on the risk of experiencing sexual harassment during residency training remains unclear. The purpose of this study was to determine the proportion of orthopaedic surgeons with sexual and gender minority identities who experience sexual harassment during training and report their experiences.

**Methods:**

An anonymous survey was distributed through Microsoft Forms to resident and active members of PrideOrtho. Respondents provided the following information: demographics, time since graduating residency, and residency program geographic location. Experiences of sexual harassment and reporting during residency were analyzed, and associations with demographic variables were examined.

**Results:**

In total, 47 survey responses were analyzed. Male trainees reported experiencing less sexual harassment compared with gender minority trainees (3 [15.0%] versus 17 [67.7%], *P* = 0.001). Reported sexual harassment instance did not differ significantly between White and minority racial groups (19 [46.3%] vs 1 [33.3%], *P* = 0.55). Only 8 (38.1%) of those who experienced sexual harassment during residency reported their experience. The most cited reason for nondisclosure was that the harasser was a supervisor (8; 61.5%).

**Conclusion:**

We found that male trainees reported markedly less sexual harassment compared with gender minority trainees. Racial or sexual minority status did not markedly influence the reported incidence of sexual harassment. Of participants who experienced sexual harassment, only one-third reported the incident, largely because of a perceived lack of support. This finding highlights the need for residency programs to implement training and support systems to limit sexual harassment and to support residents that do experience workplace harassment. Additional research is needed to better understand how the intersection of multiple minority identities may influence the experiences of orthopaedic residents regarding workplace sexual harassment.

Representation of diverse populations in orthopaedic surgery continues to lag behind other surgical specialties.^1,2^ The proportion of orthopaedic surgery trainees who identify as members of underrepresented minorities (URMs) encompassing gender, racial, ethnic, sex, and sexual orientation identities has increased over time, with an 11% increase in the number of Hispanic orthopaedic residents, a 11% increase in the number of Black orthopaedic residents, and a 27% increase in female orthopaedic residents; however, the proportion of URM faculty has changed minimally.^3^ In addition, URM orthopaedic surgeons report higher rates of discrimination in the workplace based on race and sexual orientation.^4^ URM orthopaedic surgeons report discrimination in the workplace based on race and sexual orientation.^5-7^ Furthermore, female orthopaedic surgeons also experience sexual harassment at alarming rates during their training. Whicker et al^8^ found that 68% of female orthopaedic surgery residents and attendings reported that they had experienced sexual harassment during residency training. However, the effect of having multiple URM identities during orthopaedic surgery training has not been well studied.

Although there have been efforts to improve diversity in orthopaedic surgery, the effect of these efforts on the workplace environment is not well characterized.^9-11^ A recent study of members of the Pediatric Orthopaedic Society of North America showed that women still report markedly lower scores than men when evaluating workplace culture, such as fair treatment toward women or regarding experiences of bullying.^12^ In addition, the perception of orthopaedic surgery as a “boys' club” may further affect the inclusivity felt by gender and sexual minority orthopaedic surgeons during training and in the workplace.^13-15^ Harrington et al highlighted that the critical number of 30% representation (ie, critical mass), which allows a group to become a stakeholder and have greater influence on culture, has yet to be reached by women and minority groups in orthopaedic surgery.^15^

In 2024, Freedman-Weiss et al^16^ evaluated experiences of sexual harassment among 270 general surgery residents across 16 training programs. The authors found that sexual harassment is often underreported in surgical training programs because of ineffective responses to reporting. In this study, 47.7% of general surgery residents who experienced sexual harassment indicated that they did not report their experiences because they felt it would be a waste of time. The purpose of this study was to determine the proportion of orthopaedic surgeons with sexual and gender minority identities who experienced sexual harassment during residency training and subsequently reported their experiences.

## Methods

### Survey

An anonymous 17-question voluntary online survey was distributed through Microsoft Forms to resident and active members of PrideOrtho in December 2024. A follow-up e-mail was sent 2 weeks later to encourage more participation.

The survey used in this study was adapted from a study published in 2020 by Whicker et al, investigating the proportion of female orthopaedic surgeons who have been sexually harassed during residency training.^6^ It was modified to include questions regarding the participants' identities (including sex, race, and sexuality), the perceived attitudes of their residency program, (including perceived safety in disclosing sexual identity and how the program would have responded to reported incidents), and respondents' experiences of sexual harassment during orthopaedic residency.

### Study Population

The anonymous survey was distributed to all active and resident members of PrideOrtho by staff affiliated with this organization. PrideOrtho is a group that aims to diversify the orthopaedic community and support the professional development of orthopaedic surgeons from all backgrounds who identify as LGBTQ+. Members of PrideOrtho are part of the LGBTQ+ community identifying as lesbian, gay, bisexual, transgender, queer, or allies, aligning with the mission of the organization.

### Primary and Secondary Study End Points

Our primary study end point was to determine the proportion of gender and sexual minority orthopaedic surgeons who experienced sexual harassment during residency training. Our secondary study end points were to assess the proportion of trainees who reported these experiences, further characterize the discrepancy in incident reporting, and determine whether there has been a change in the proportion of recent gender and sexual minority orthopaedic trainees who experience sexual harassment during training relative to attendings and fellows who had already completed residency.

### Statistical Analysis

Descriptive and comparative statistics were used to analyze survey responses. Categorical variables including gender, race, sexual identity, year in training, and whether respondents experienced or reported sexual harassment were compared using Chi-squared tests for with a predetermined significance of *P* < 0.05. Odds ratios with a 95% confidence interval (CI) were also calculated to estimate the effect of respondent demographics on the risk of experiencing sexual harassment during residency. Haldane-Anscombe correction was conducted where a categorical value was 0. Quantitative data describing individual experiences were also collected but were not amenable to analysis. All analyses were conducted using Microsoft Excel (Supplementary Data File, http://links.lww.com/JG9/A524).

## Results

### Study Population

In total, 51 surveys were completed, four of which were excluded because the respondents were medical students or completed residency in specialties other than orthopaedic surgery. Nine respondents (19.2%) were current residents. The greatest participation came from physicians who had completed residency 1 to 5 years ago with 13 responses (27.7%), while the smallest participation came from physicians who completed residency 20 to 30 years ago with only one 1 response (2.1%). Twenty-two of 47 respondents (47%) identified their sexual orientation as heterosexual, 14 (30%) identified their sexual orientation as gay or lesbian, and 8 (17%) identified their sexual orientation as bisexual (Table 1). In addition, 25 (53.2%) were female, 20 (42.5%) were male, and 2 (4.3%) identified as a gender minority (Table 1). Regarding year in training/practice, 9 respondents (19%) were current residents while 38 (81%) were in practice. Each geographic region within the United States was represented by the respondents. The largest number of respondents was from the Northeast and Pacific regions, while the fewest number of respondents was from the Midwest (Table 1).

**Table 1:** Participant Identities and Training Level

Questions About Identity and Residency Training	Total, % (N = 47)	Harassed, %
Sex		
Female	25 (53.2)	17 (68.0)
Male	20 (42.6)	3 (15.0)
Gender minority	2 (4.3)	1 (50.0)
Race		
White	41 (87.2)	19 (46.3)
Black	3 (6.4)	1 (33.3)
Asian	2 (4.3)	1 (50.0)
Alaska Native/Native American	1 (2.1)	0 (0.0)
Sexual identity		
Heterosexual	22 (46.8)	9 (40.9)
Gay/lesbian	14 (29.8)	7 (50.0)
Bisexual	8 (17.0)	3 (37.5)
Queer	1 (2.1)	0 (0.0)
Prefer not to say	2 (4.3)	2 (100.0)
Felt residency would be a safe place to disclose sexual identity		
Yes	24 (51.1)	8 (33.3)
No	9 (19.2)	7 (77.8)
Unsure	14 (29.8)	6 (42.9)
Year in training		
Current resident	9 (19.2)	3 (33.3)
Completed residency 1-5 yr	13 (27.7)	5 (38.5)
Completed residency 5-10 yr	10 (21.3)	6 (60.0)
Completed residency 10-15 yr	3 (6.4)	2 (66.7)
Completed residency 15-20 yr	5 (10.6)	3 (60.0)
Completed residency 20-30 yr	1 (2.1)	0 (0.0)
Completed residency >30 yr	6 (12.8)	2 (33.3)
Region of orthopaedic surgery residency program		
Midwest	**9 (19.2)**	**4 (44.4)**
East North Central	5 (10.6)	2 (40.0)
West North Central	4 (8.5)	2 (50.0)
Northeast	**16 (34.0)**	**10 (62.5)**
Mid-Atlantic	8 (17.0)	6 (75.0)
New England	8 (17.0)	4 (50.0)
South	**11 (23.4)**	**2 (18.2)**
South Atlantic	5 (10.6)	1 (20.0)
West South Central	6 (12.8)	1 (16.7)
West	**11 (23.4)**	**5 (45.5)**
Mountain	3 (6.4)	1 (33.3)
Pacific	8 (17.0)	4 (50.0)

### Overall Proportion of URMs Reporting Sexual Harassment During Residency Training

Seventeen of 25 female respondents (68%) reported experiencing sexual harassment during their orthopaedic surgery training. Male orthopaedic trainees reported experiencing less sexual harassment compared with female and gender minority orthopaedic trainees (three of 20 [15.0%] versus 18 of 27 [67.7%], odds ratio 11.33 [95% CI, 2.6 to 49.1]; *P* value = 0.001). The number of disclosed instances of sexual harassment was not significantly different between White and minority racial groups (19 of 41 [46.3%] versus two of six [33.3%], odds ratio 0.6 [95% CI, 0.1 to 3.5]; *P* = 0.55; Tables 1 and 2).

**Table 2:** Statistical Significance of Reported Sexual Harassment

Reporting Sexual Harassment		Odds Ratio	95% CI	*P* Value
Sex				
Male	Female + gender minorities			
15.0% (three of 20)	67.7% (18 of 27)	11.3	2.6-49.1	<0.001
Race				
White	Person of color			
46.3% (19 of 41)	33.3% (two of six)	0.6	0.1-3.5	0.549
Sexual orientation				
Heterosexual	Sexual minority			
40.9% (nine of 22)	48.0% (12 of 25)	1.3	0.4-4.2	0.626
Heterosexual	Bisexual			
40.9% (nine of 22)	37.5% (three of eight)	0.9	0.2-4.6	0.866
Heterosexual	Gay/lesbian			
40.9% (nine of 22)	50.0% (seven of 14)	1.4	0.4-5.6	0.593
Heterosexual	Queer			
40.9% (nine of 22)	0.0% (zero of one)	0.5	0.0-12.9	0.412
Heterosexual	Prefer not to say			
40.9% (nine of 22)	100.0% (two of two)	7.1	0.3-165.5	0.108
Training				
Current trainees	Past trainees			
33.3% (three of nine)	47.4% (18 of 38)	1.8	0.1-2.6	0.446
Felt safe disclosing sexual identity	Felt unsafe disclosing sexual identity			
33.3% (eight of 24)	77.8% (seven of nine)	7.0	1.2-41.8	0.022
Felt safe disclosing sexual identity	Felt unsure disclosing			
33.3% (eight of 24)	42.9% (six of 14)	1.5	0.4-5.8	0.558
Geography				
New England	South			
62.5% (ten of 16)	18.2% (two of 11)	0.1	0.0-0.8	0.022
New England	Mid-West			
62.5% (ten of 16)	44.4% (four of nine)	0.5	0.1-2.5	0.383
New England	West			
62.5% (ten of 16)	45.5% (five of 11)	0.5	0.1-2.4	0.381

CI = confidence interval

### Proportion of Trainees Experiencing Sexual Harassment Who Reported the Incident While in Training

While 21 respondents disclosed experiencing sexual harassment, only 8 (38.1%) reported the incident to people within their training institution and 13 respondents (61.9%) offered reasons of not reporting their experience within their training environment (Table 2). The most common reason for not reporting the experienced sexual harassment was that “the harasser was my superior (eg, resident and attending),” with eight of 13 respondents (61.5) endorsing this as “the most important reason” for not reporting. Four of 13 respondents (30.8%) indicated that they believed that the department would not act on their report, and that this was “the most important reason” for not reporting (Figure 1).

**Figure 1:**
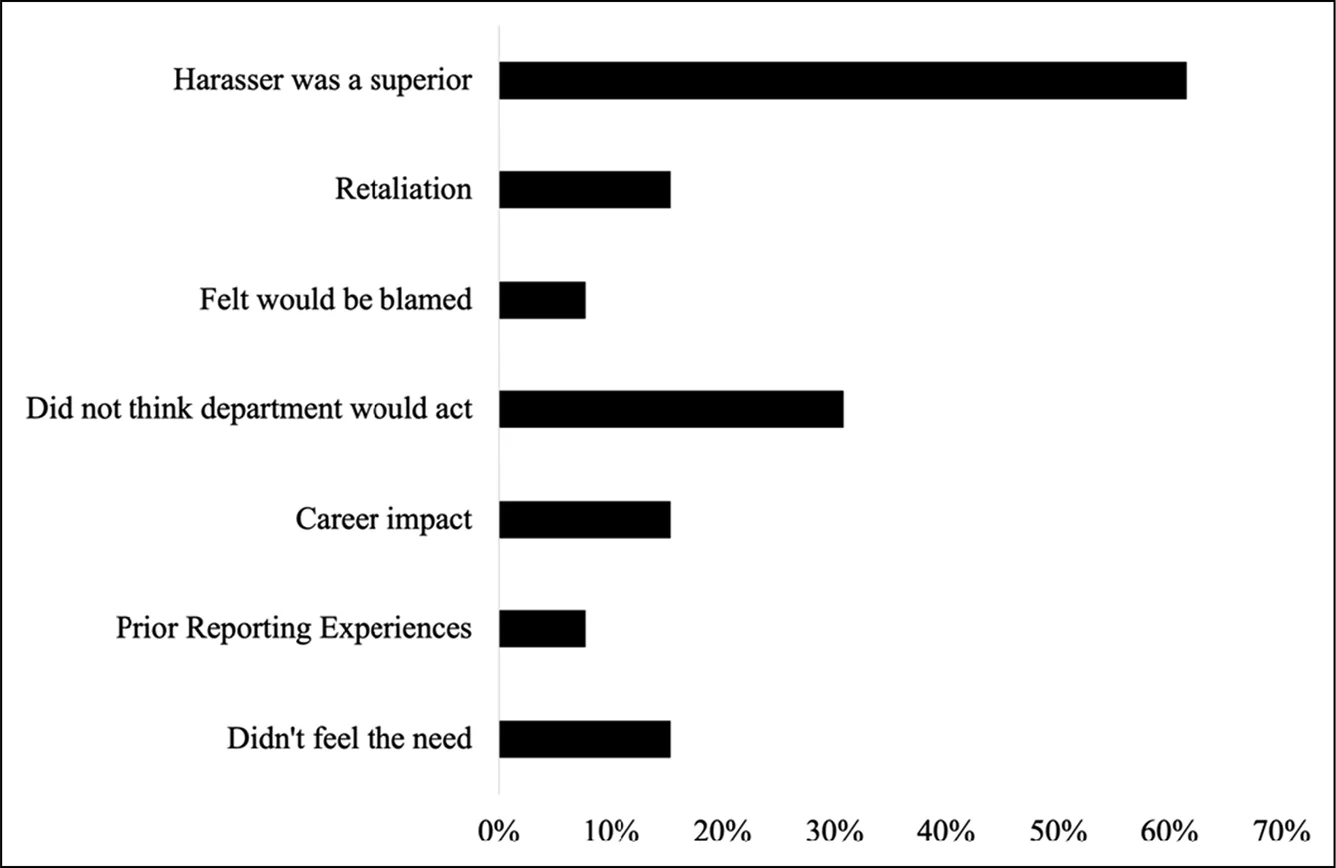
Chart demonstrating reasons cited by residents for not reporting sexual harassment.

### Additional Findings

In total, 24 of 47 (51%) felt that their orthopaedic surgery residency program would be a safe place for LGBTQ+ identities to be disclosed. However, respondents who did not feel safe disclosing their sexual orientation had significantly higher odds of reporting sexual harassment (seven of nine [77.8%] versus eight of 24 [33%], odds ratio 7.0 [95% CI, 1.2 to 41.8], *P* = 0.022). The most frequent type of sexual harassment disclosed was “verbal remarks” (20 of 21; 95.2%; Figure 2). In addition, 17 participants (81.0%) reported experiencing multiple types of harassment. In 15 incidences (71.4%), the perpetrators of sexual harassment were reported to be performed by male residents, and these incidences included two reports of sexual assault (Figures 2 and 3).

**Figure 2:**
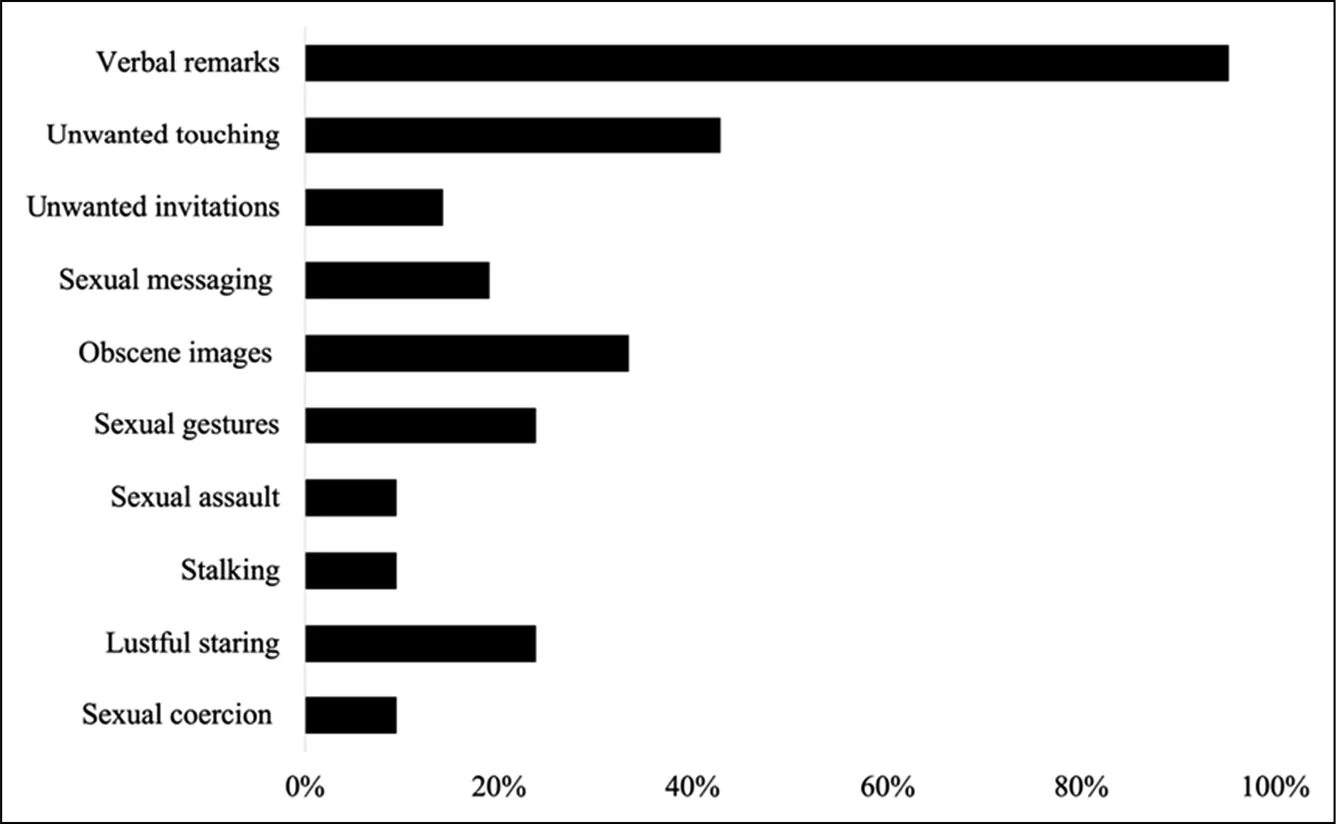
Chart demonstrating types of sexual harassment experienced by respondents during residency.

**Figure 3:**
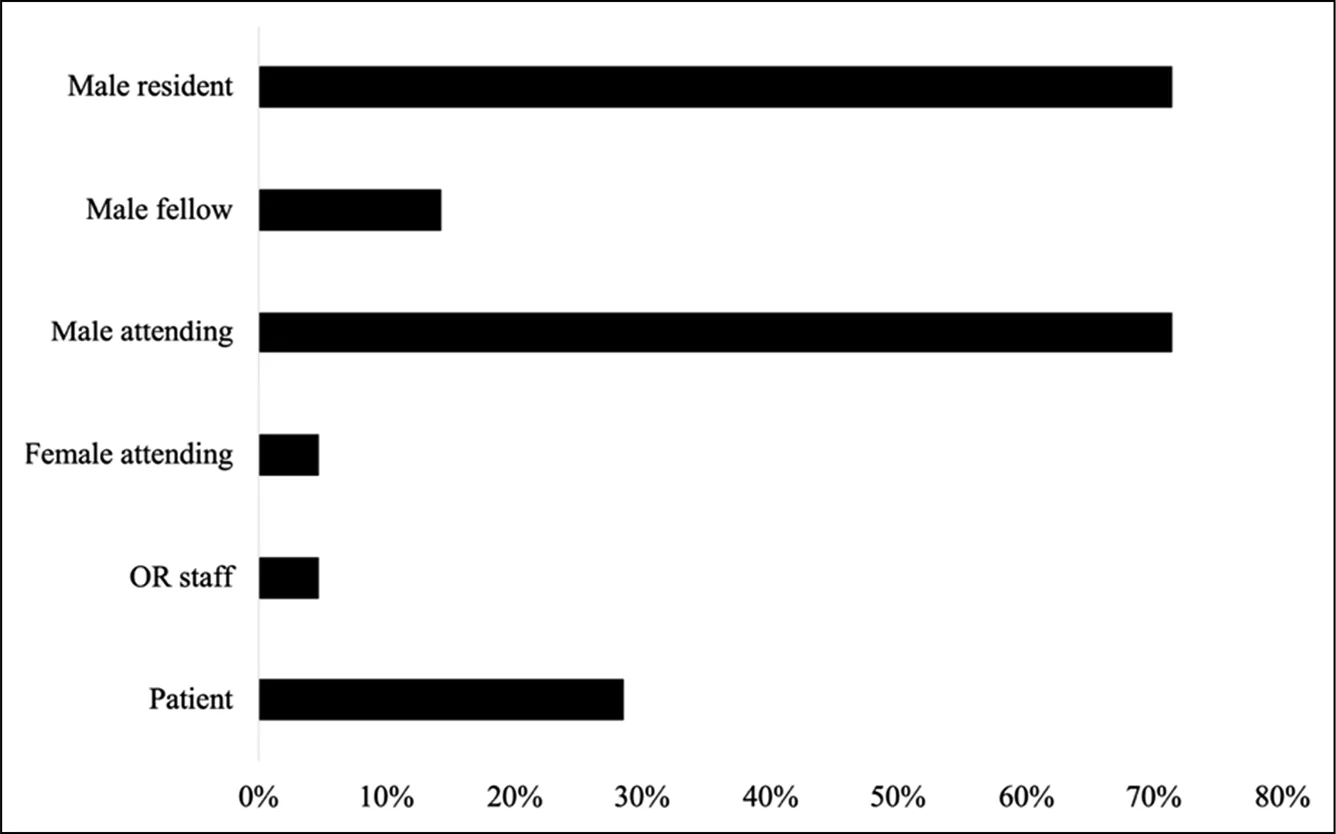
Chart demonstrating identity of alleged sexual harassers.

### Proportion of Current versus Past Trainees Reporting Sexual Harassment

No difference was observed in the proportion of reported occurrences of sexual harassment by current trainees and past trainees in orthopaedic surgery residency training (three of nine [33.33%] versus 18 of 38 [47.37%], odds ratio 0.56 [95% CI, 0.12 to 2.55]; *P* = 0.45).

### Qualitative Data of Sexual Harassment Experiences

Sixteen of 21 respondents (76%) who reported experiencing sexual harassment during their training elaborated on their specific experiences in their own words (Table 3).

**Table 3:** Specific Allegations of Sexual Harassment Provided by Respondents (Warning: Please Note That Table 3 Contains Sexually Explicit Language)

Residency Status	Allegation
Current resident	“Being asked about my sex life by an anesthesiologist and none of my teammates said anything. A naked trauma patient on the OR table and a senior resident commented how “hot” she was
0 to 5 yr ago	“My co resident got drunk and would not stop touching me at a party with other residents/other people. I pushed and shoved him away and he would not stop and everyone just watched and laughed as I struggled”
0 to 5 yr ago	“Miming sexual intercourse during operations particularly during canal preparation or long nail positioning”
0 to 5 yr ago	“My attending asks me directly when we would have sex, after 2 days of new hospital assignment. I saw attendings harass nurses and female residents pretending it was a joke. I saw a lot of colleagues (male and female) telling the victims to pretend nothing happened or not to complain because it is normal in the orthopedic world”
6 to 10 yr ago	“Attending said that because I was gay, that I probably excelled at fellatio and had other sexual skills, in front of a group of other attendings. Another attending said that I didn't ‛look gay.’ Another attending made remarks about what they thought I did sexually with others”
6 to 10 yr ago	“Asking jokingly if I was interested in oral sex on them or to not stand behind them as they suggested I might force anal sex on them. Jokes were often made when I was not in the room about sexual position or what kind of people I had sex with”
11 to 15 yr ago	“Likely was drugged by a senior resident (no other explanation for being in-and-out of consciousness) and sexually assaulted. I would have been blamed b/c I had been drinking with the residents”
16 to 20 yr ago	“Male resident sitting with me in a small room doing dictations after clinic, leaning back in the chair rubbing his nipples while talking to me Male attending brushing his hand past my rear end several times in clinic”
>30 yr ago	“Derogatory comments about women's body parts, sexual analogies instructions in sexual terms ‛rub it like you love it,’ etc. staring, elbowing in the boobs, calendars, and nudies in call rooms and conferences”

OR – operating room

## Discussion

The purpose of this study was to determine the proportion of gender and sexual minority orthopaedic surgeons who experienced sexual harassment during residency training and subsequently reported their experiences. We found that male trainees reported markedly fewer incidents of sexual harassment compared with gender minority trainees. Notably, racial or sexual minority status alone was not associated with a statistically significant difference in reported harassment compared with White or heterosexual respondents. The most frequently reported form of harassment was verbal remarks (95.2%), and most respondents experienced multiple forms of harassment, often perpetrated by male residents. Among those who experienced sexual harassment, only approximately one-third reported the incident, largely because of a perceived lack of support.

While 21 respondents disclosed experiencing sexual harassment, only eight reported the incident to someone within their training institution and 13 respondents offered reasons for not reporting their experience within their training environment. The leading indicated reason for not reporting the experienced sexual harassment was that “the harasser was my superior (eg, resident and attending),” with 61.5% (eight of 13) of respondents endorsing this as “the most important reason” for not reporting, highlighting the potential for unsafe work environments in residency training due to hierarchy. Similarly, Gianakos et al^17^ examined the prevalence and effects of bullying, discrimination, harassment, and sexual harassment on the well-being and productivity of surgical residents.^12^ The authors found that 27% (5793/19,631) experienced sexual harassment and that attending surgeons were the most common perpetrators.

Experiences of harassment among female and gender and sexual minority orthopaedic surgeons and distrust in the workplace to respond appropriately to harassment reports do not end with the completion of residency. Balch Samora et al^18^ evaluated experiences of workplace discrimination, bullying, sexual harassment (DBSH), and harassment among orthopaedic surgeons in practice. The authors found that 66% (612/927) of female and URM members of the American Academy of Orthopaedic Surgeons experienced DBSH, with 47% (288/612) reporting sexual harassment specifically. Furthermore, the perceived confidence in the workplace to effectively deal with DBSH behaviors was only 58% (538/927).

In our study, 17 of 25 female respondents (68%) reported experiencing sexual harassment during their orthopaedic surgery training. Similarly, Whicker et al^8^ examined the proportion of women having experienced sexual harassment during orthopaedic surgery residency training. The authors found the same proportion of women reporting having experienced sexual harassment (171 of 250, 68%). We found that male orthopaedic trainees reported experiencing less sexual harassment compared with female and gender minority orthopaedic trainees (15.0% versus 67.7%). While male trainees reported experiencing less sexual harassment than female trainees, this may be from hesitancy to label their experiences as harassment.^19^ The number of disclosed sexual harassment incidents was not significantly different between White and minority racial groups in this cohort of responses from PrideOrtho (46.3% versus 33.3%).

Sexual harassment experienced by respondents went largely unreported to their training programs. Approximately 31% of respondents indicated that they believed that the department would not act on their report and that this was “the most important reason” for not reporting (Table 4). This lack of confidence in programs' responses to reported sexual harassment is mirrored in other studies. Freedman-Weiss et al^16^ evaluated the occurrence and reporting of sexual harassment in general surgery residents. The authors found that 47% of general surgery residents in training considered reporting the harassment to be “a waste of time.”

**Table 4:** Characteristics of Reported Sexual Harassment

Sexual Harassment Characteristics	Total, % (N = 47)
Sexual harassment	
Yes	21 (44.7)
No	26 (55.3)
Race of perpetrator	
Same race	15 (71.4)
Different race	4 (19.1)
Unknown	2 (9.5)
Reported incident in medicine	8 (38.1)
Co-resident	4 (19.1)
Attending	2 (9.5)
Chairman	1 (4.8)
Program director	1 (4.8)
Faculty outside orthopedics department	1 (4.8)
Reported outside of medicine	12 (57.1)
Family	2 (9.5)
Friend	8 (38.1)
Therapist	1 (4.8)
Mentor	1 (4.8)
Attorney	1 (4.8)

There are several limitations to this study. First, owing to the sensitive nature of the topic being studied, the number of respondents to the survey was relatively low. In addition, this study only surveyed resident and active members of PrideOrtho and, as such, inherently introduced selection bias and may not be applicable to other URM groups. Third, asking attending surgeons, fellows, and residents to reflect on experiences of sexual harassment during residency subjects their responses to recall bias. Fourth, definitions of sexual harassment may vary between respondents and, therefore, influence reporting in this survey. While some of the types of sexual harassment reported in this study have clear definitions, such as “stalking,” other reported harassment was subject to the respondents' personal definition, such as what words qualified as offensive “verbal remarks.”

## Conclusion

We found that male trainees reported experiencing markedly less sexual harassment compared with gender minority trainees. Racial or sexual minority status did not markedly influence the reported incidence of sexual harassment. Of participants who experienced sexual harassment, only one-third reported the incident, largely because of a perceived lack of support. This finding highlights the need for residency programs to implement training and support systems to limit sexual harassment and to support residents that do experience workplace harassment. Additional research is needed to better understand how the intersection of multiple minority identities may influence the experiences of orthopaedic residents regarding workplace sexual harassment.

## Supplementary Material
